# Crown architecture traits mediate shade tolerance-dependent trade-offs and neighborhood interactions in a subtropical forest

**DOI:** 10.1016/j.pld.2026.02.004

**Published:** 2026-02-09

**Authors:** Qi Wu, Xingchen Wang, Yumeng Huang, Chengwei Li, Lisheng Yang, Zijun Zeng, Yishan Shi, Jianhua Chen, Yunquan Wang

**Affiliations:** a College of Life Sciences, Zhejiang Normal University, Jinhua 321004, China; b Zhuji Natural Resources and Planning Bureau, Shaoxing 311800, China; c State Key Laboratory for Vegetation Structure, Function and Construction, College of Life Sciences, Zhejiang University, Hangzhou 310058, China; d The Administration Center of Zhejiang Jiulongshan National Nature Reserve, Lishui 323300, China

**Keywords:** Crown architecture, Functional trade-off, Light competition, Neighborhood interactions, Shade tolerance, Subtropical forest

## Abstract

Crown architectural traits are critical adaptations that balance light capture with mechanical stability. Given that light availability plays a fundamental role in shaping forest community structure, co-occurring tree species of different shade tolerance guilds exhibit distinct resource acquisition strategies. However, how shade tolerance governs multidimensional crown architecture and mediates neighborhood interactions remains unclear. In a subtropical Chinese forest, we monitored 5-year growth and measured six individual-level crown traits for 3589 trees. We quantified trade-offs among crown traits and evaluated the relative effects of tree size, spatial structure, neighborhood density, and crown trait dissimilarity on growth across shade tolerance guilds. Principal component analysis revealed two major axes: crown shape (PC1, narrow-deep vs. broad-shallow) and crown size (PC2, height and apical dominance). Light-demanding species exhibited higher scores along the crown size axis, consistent with a strategy of rapid vertical growth, whereas shade-tolerant species showed more plastic crown forms, advantageous for persistence under low light conditions. The influence of crown trait-mediated neighborhood interactions on growth also diverged between shade tolerance guilds. Growth of light-demanding species was suppressed by dissimilarity in apical dominance ratio and crown shape (PC1), yet enhanced by dissimilarity in crown projection area, reflecting both environmental filtering and niche differentiation. In contrast, shade-tolerant species were mainly constrained by conspecific density, consistent with density dependent effects potentially linked to natural enemies. These findings demonstrate that shade tolerance structures crown trait trade-offs and determines how crown traits mediate neighborhood interactions, thereby improving our understanding of crown architecture-driven demography and coexistence in forest ecosystems under global change.

## Highlights

• Crown traits vary along crown shape (narrow-deep vs. broad-shallow) and crown size (height and apical dominance) axes.
• Light-demanding species exhibit higher scores on crown size axis, characterized by taller and narrower crowns that enhance light interception.
• Trait dissimilarity promotes growth in light-demanding species, whereas growth in shade-tolerant species is limited by conspecific density.

## 1 Introduction

Trees exhibit diverse crown architectures in response to biotic and abiotic environments (Nakamura et al., 2017; Wu et al., 2025; Jucker et al., 2025). Mirroring trade-offs observed in organ-level traits such as specific leaf area and wood density (Joswig et al., 2021; Maynard et al., 2022), crown architectural traits also exhibit coordinated functional adaptations. These adaptations optimize light interception efficiency and hydraulic performance across varying climatic regimes (Duchesneau et al., 2001; Su et al., 2020). Given that crown architecture is highly responsive to resource availability and spatial heterogeneity, growing empirical evidence highlights its role in mediating neighborhood interactions and shaping community structure (Hajek et al., 2015; Pescador et al., 2019). However, how crown-mediated interactions regulate growth dynamics remains poorly understood within trait-based framework (Laurans et al., 2024).

Plant functional traits integrate morphological, physiological, and structural attributes to influence plant fitness (Violle et al., 2007; Walker et al., 2022; Liu et al., 2024b). Woody plant ecological strategies are often described along two orthogonal trait axes (Díaz et al., 2016; Rüger et al., 2020): (1) the “fast–slow” economics spectrum, which reflects trade-offs between growth rate and survival (Wright et al., 2004, 2010; Reich, 2014); and (2) the “stature–recruitment” trade-off, which balances competitive advantage through height against resource allocation to reproduction (Kohyama, 1993; Guillemot et al., 2022; Kambach et al., 2022). However, current trait-based frameworks primarily reduce crown architecture to simplistic proxies such as tree height, which capture only vertical light competition and overall size (e.g., Verbeeck et al., 2019; Xu et al., 2020; Maynard et al., 2022; McNeil et al., 2023). This simplification overlooks the multidimensional trade-offs inherent in crown architecture. Crown architectures represent integrated adaptations that optimize light interception through traits such as crown size, while maintaining mechanical stability under environmental stresses such as wind loading (MacFarlane and Kane, 2017; Maharjan et al., 2021). Moreover, intraspecific plasticity along environmental gradients contributes to additional variation in crown allometry (Pearcy et al., 2005; Jackson et al., 2019; Liu et al., 2024a), suggesting that the unmeasured crown traits may represent distinct dimensions of ecological strategy.

Trait-mediated neighborhood interactions are central but understudied drivers of demography and ecosystem function (Lasky et al., 2014; Kunstler et al., 2016; Zhang et al., 2024). Functional dissimilarity among neighbors could reduce competition through niche differentiation (Limiting-similarity hypothesis; Macarthur and Levins, 1967; Wagg et al., 2017). Conversely, environmental filtering may promote trait convergence and suppress individual performance (Qian et al., 2022; Wang et al., 2024; Nevins and Zambrano, 2024). Notably, the majority of empirical evidence to date has focused on organ-level traits as predictors of interaction outcomes (Lasky et al., 2014; Kunstler et al., 2016; Nemetschek et al., 2025), despite well-documented relationships between crown architectural metrics (e.g., crown projection area) and resource acquisition strategies (Liu et al., 2016). Recent studies demonstrate that integrative traits reflecting crown allocation can improve the accuracy of demographic predictions (Yang et al., 2018, 2021; Rubio et al., 2021). Crown architecture also influences plant interactions by facilitating physical niche differentiation (e.g., vertical stratification) and altering the local the light microenvironment (Kunz et al., 2019; Ma et al., 2023; Beauchamp et al., 2025). However, these mechanisms remain insufficiently integrated into current functional ecology frameworks.

**Fig. 1:**
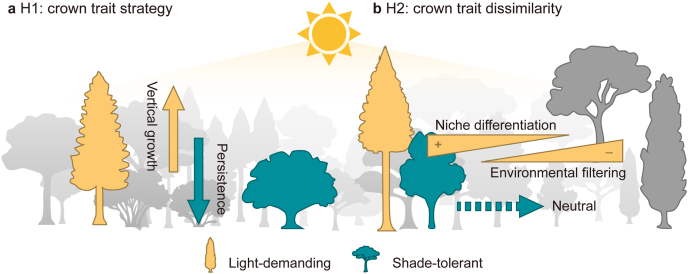
**Conceptual framework based on the priori hypotheses of this study.** (a) Light-demanding species are expected to develop taller and narrower crown architectures that facilitate vertical growth, whereas shade-tolerant species are predicted to adopt crown structures that enhance persistence under low-light understory conditions. (b) Growth performance in light-demanding species is anticipated to be more strongly influenced by crown trait dissimilarity, reflecting intense competition for light. Functional dissimilarity may reduce competition pressure through niche differentiation, while environmental filtering may promote trait convergence and thereby constrain individual performance. In contrast, shade-tolerant species are expected to be less sensitive to crown trait dissimilarity among neighbors.

Light availability is a critical environmental factor shaping community structure and species coexistence (Sterck et al., 2011; Onoda et al., 2014). Species exhibit a wide range of ecological strategies for light capture and utilization, giving rise to a continuous spectrum of shade tolerance (Valladares and Niinemets, 2008; Ouédraogo et al., 2013). At the conservative end of this spectrum, shade-tolerant species are characterized by slow growth, compact stature, and traits that favor persistence under low-light conditions (e.g., high leaf mass per area; Kunstler et al., 2009; Poorter, 2009). Conversely, light-demanding species prioritize rapid height growth and canopy dominance, but at the cost of increased vulnerability to light competition from neighboring individuals (Kobe and Vriesendorp, 2011; Chen et al., 2019b). Based on this functional contrast, we propose the following hypotheses (Fig. 1): (1) Crown architecture reflects multidimensional trade-offs that vary with shade tolerance (H1). Light-demanding species are expected to show taller and narrower crown architectures that maximize vertical growth, whereas shade-tolerant species are predicted to adopt crown structures that enhance persistence in shaded understory environments. (2) The influence of crown trait dissimilarity on tree growth varies across shade-tolerance guilds (H2). Growth performance in light-demanding species is predicted to be more strongly influenced by crown trait dissimilarity due to intense competition for light, compared to shade-tolerant species.

To address these theoretical predictions, we analyzed 5-year growth data from 3589 trees representing 31 species, along with individual-level crown architectural data, collected in the Dongbaishan Forest Dynamics Plot. To examine H1, we examined how crown architecture is structured by multidimensional trade-offs and whether these trade-offs vary with shade tolerance guilds. Specifically, we quantified six crown traits for all individuals, applied principal component analysis (PCA) to identify major axes of variation, and used correlation analyses and non-parametric tests to assess differences between shade-tolerance guilds. To test H2, we evaluated how the relative importance of crown trait dissimilarity in driving growth rates differs between light-demanding and shade-tolerance species. We calculated neighborhood crowding indices based on crown trait dissimilarity, incorporated conspecific neighborhood density and spatial covariates, and fitted linear mixed-effects models to evaluate growth responses across shade-tolerant guilds.

## 2 Materials and methods

### 2.1 Study site

The study was conducted in a 1-ha forest dynamics plot (100 m × 100 m; 29°34′03.62″N, 120°23′38.16″E; elevation 135–157 m a.s.l.) located within the Dongbaishan Nature Reserve, Zhejiang Province, China. The region has a humid subtropical climate with average annual temperature of 11.7 °C and mean annual precipitation of 1541.4 mm (Chai et al., 2023). The Dongbaishan Forest Dynamics Plot was established in 2013 following CForBio and ForestGEO protocols (Feng et al., 2016; Davies et al., 2021). All woody stems ≥ 1 cm diameter at breast height (DBH) were tagged, mapped, measured, and identified to species. Survival status was assessed, and new recruits were tagged, measured, mapped and identified during each 5-year intervals.

### 2.2 Crown traits

Crown architecture was quantified in 2013 using telescoping height measuring poles. Tree height (TH), crown base height (CBH), as well as the crown widths in the east-west (Ca) and north-south (Cb) directions were measured for each tagged woody individual. Six crown architectural traits were derived following established methodologies to represent crown architecture (Table 1; Li et al., 2017; Martin-Ducup et al., 2020; Su et al., 2020): tree height (TH), crown depth (CD), live crown ratio (LCR), crown aspect ratio (CAR), apical dominance ratio (ADR) and crown projection area (CPA). Data containing obvious anomalies in crown or DBH records were excluded, resulting in a final dataset comprising 3589 individuals from 31 species.

**Table 1:** **Crown functional traits quantified in the Dongbaishan forest.** Traits were derived from field measurements of tree height (TH), crown base height (CBH), and crown widths (east-west: Ca, and north-south: Cb). Mathematical formulas and corresponding units are specified for each crown trait.

Trait	Formula	Ecological Interpretation	Reference
Tree height (TH, m)	Measured	Trade-off between light competition and hydraulic vulnerability; vertical growth strategy.	Givnish et al. (2014)
Crown depth (CD, m)	TH − CBH	Balance between photosynthetic capacity and structural resilience to abiotic stressors.	Vermeulen (2014)
Live crown ratio (LCR)	CD/TH	Resource allocation to photosynthetic tissues relative to vertical height investment.	Hasenauer and Monserud (1996)
Crown aspect ratio (CAR)	0.5 × (Ca + Cb)/CD	Crown shape adaptation balancing light interception and hydraulic safety.	Orman et al. (2023)
Apical dominance ratio (ADR)	TH/[0.5 × (Ca + Cb)]	Vertical growth prioritization over lateral expansion; light competition and meristem reserve strategy.	Aarssen (1995)
Crown projection area (CPA, m^2^)	π × Ca × Cb	Horizontal light capture potential; spatial dominance in canopy layer.	Nilson (1999)

### 2.3 Neighborhood variables

To assess the neighborhood effects mediated by crown traits while accounting for density dependence, we calculated the neighborhood crowding index of trait dissimilarity (NCIS) and the density of conspecific and heterospecific neighbors (Neighborhood density) following Lasky et al. (2014) and Han et al. (2025):

(1) $$\begin{array}{c} {NCIS}_{i}=\sum_{j=1,i\neq j}^{j}\left(\left|F_{i}-F_{j}\right|\frac{{{DBH}_{j}}^{2}}{{d_{ij}}^{2}}\right) \end{array}$$

(2) $$\begin{array}{c} {Neighborhood\;density}_{i}=\sum_{j=1,i\neq j}^{j}\left(\frac{{BA}_{j}}{d_{ij}}\right) \end{array}$$

where $F_{i}$ and $F_{j}$ are the trait values for the focal individual *i* and the neighboring individual *j* within the local neighborhood scale, ${DBH}_{j}$ is the DBH of neighbor *j,* $d_{ij}$ is the distance between the focal individual *i* and the neighboring individual *j* (Uriarte et al., 2004; Kunstler et al., 2012), and ${BA}_{j}$ refers to the basal area of conspecific or heterospecific neighboring individual *j*. The absolute trait difference term ($\left|F_{i}-F_{j}\right|$) indicates that more intense neighborhood interactions are associated with greater dissimilarity in functional traits.

Neighbors were defined as all individuals within a 10-m radius of each focal tree, a spatial scale shown to effectively capture competitive and facilitative interactions in forest ecosystems (Thorpe et al., 2010). A total of 2429 focal individuals from 12 species were selected, with the criteria that each was located least 10 m away from the plot edge and had a minimum population abundance of five individuals per species (Chen et al., 2019b).

### 2.4 Growth rate

To investigate how crown traits along with their dissimilarity correlates with growth performance, we quantified the annual growth rate (AGR, cm^2^/year) of basal area as follows formula:

(3) $$\begin{array}{c} AGR=\frac{\pi }{t_{2}-t_{1}}\times \left({\left(\frac{{DBH}_{t_{2}}}{2}\right)}^{2}-{\left(\frac{{DBH}_{t_{1}}}{2}\right)}^{2}\right) \end{array}$$

where *t*_2_ and *t*_1_ refer to the measurement years 2018 and 2013, respectively (Stoll et al., 1994). Negative AGR values, which indicate stem shrinkage, were excluded from the analysis. A constant value of 0.01 was added to the AGR before log-transforming to avoid computational issues with zero or near-zero values.

### 2.5 Shade tolerance guilds

To account for interspecific variations in the ecological strategies related to light capture and utilization (Touzot et al., 2025), we categorized species into shade tolerance guilds based on the “light figure” compiled by Song et al. (2013). This index reflects the relative light intensity of the habitats in which a species typically occurs. Species with a light figure greater than 6 were categorized as light-demanding, typically occurring in sparse stands, forest edges, or open habitats, whereas those with a value of 6 or less were considered shade-tolerant, and generally found under dense forest canopies. Representative examples species include *Pinus massoniana* (light figure = 9) as light-demanding species and *Schima superba* (light figure = 6) as shade-tolerant species (see details in Table S1). Overall, light-demanding species accounted for 35.7% of stems and 51.6% of total basal area, while shade-tolerant species accounted for 64.3% of stems and 48.4% of total basal area.

### 2.6 Statistical analysis

To address H1, we employed principal component analysis (PCA) and Spearman’s rank correlation analysis to determine different dimensions of crown trait trade-offs. Specifically, crown traits were included as active variables in the PCA, and AGR as a supplementary variable. The first principal component (PC1) and the second principal component (PC2) were considered as potential axes representing major trade-off axes (Díaz et al., 2016; Rüger et al., 2020). Spearman’s rank correlation analysis was used to examine the relationships between various crown traits and their trade-off axes associated with growth performance. Additionally, partial Spearman’s correlations were performed while controlling for log-transformed DBH to account for size-dependent effects on trait relationships. To analyze whether crown trade-off strategies differ across shade tolerance guilds, Wilcoxon’s rank-sum test were applied to compare PC1 and PC2 scores between light-demanding and shade-tolerant species.

To address H2, we used linear mixed effects models (LMMs) to examine whether the effects of crown trait dissimilarity on growth rate differ with shade tolerance guilds. To assess the influence of crown-mediated neighborhood effects on individual growth while accounting for potential confounding effects, we modeled basal area growth (AGR) of individual *k* of species *j* in plot *i* as a function of DBH, neighborhood density, spatial structure, and crown trait dissimilarity:

(4) $$AGR=\beta_{0}+\beta_{1}\times \ln\;\left({DBH}_{ijk}\right)+\beta_{2}\times {ConS}_{ijk}+{\;\beta }_{3}\times {HetS}_{ijk}+\beta_{4}\times {PCNM}_{ijk}+\beta_{5}\times \ln\;\left({NCIS}_{ijk}\right)+\varphi_{i}+\varphi_{j}$$

Here, we included three types of covariates: 1) log-transformed DBH to account for size effects, 2) conspecific and heterospecific neighborhood density to control for density dependence effects (Chen et al., 2019a; Wang et al., 2020), and 3) the first two axes from principal coordinate analysis of neighborhood matrices (PCNM) to represent spatial structure (Legendre et al., 2009; Arantes et al., 2018). PCNM variables were computed by constructing a Euclidean distance matrix among all 20 m × 20 m plots. Only eigenvectors associated with positive eigenvalues were retained as spatial predictors to capture spatial autocorrelation. We further incorporated crown trait dissimilarity (i.e., log-transformed NCIS weighted by six crown traits and PC1/PC2 scores) as explanatory variable in the models (Nemetschek et al., 2025). Species identity ($\varphi_{j}$) and plots ($\varphi_{i}$) was included as random effects to account for species-specific responses and spatial autocorrelation (Wang et al., 2020).

Our model selection and multi-model inference followed information theoretic approach (Symonds and Moussalli, 2011). We first performed all-subsets regression to evaluate all possible combinations of explanatory variables and retained the best-fit models with the lowest Akaike’s Information Criterion corrected for small sample sizes (AICc; ΔAICc < 2 threshold) for subsequent analyses (Burnham and Anderson, 2004). Model averaging was then conducted for the best-fit models to estimate the parameter coefficients for the most supported predictor set. Separate modeling procedures were constructed for shade-tolerant and light-demanding species. Where model-averaged results identified significant explanatory variables, we further assessed their robustness by checking the models with lowest predictors. All continuous variables were standardized by subtracting the mean and dividing by the standard deviation prior to analysis.

All statistical analyses and visualizations were conducted in R v.4.3.1 (R Core Team, 2024). PCA was performed using the *FactoMineR* package (Lê et al., 2008). LMMs were fitted with the *glmmTMB* package (Brooks et al., 2017). All-subsets regression and model averaging were carried out using the *MuMIn* package (Bartoń, 2013).

## 3 Results

### 3.1 Crown trait trade-off strategies of shade tolerance guilds

**Fig. 2:**
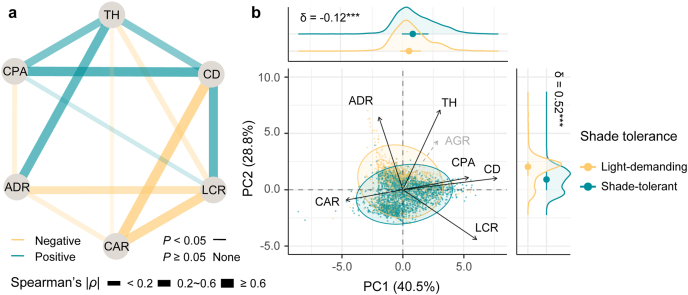
**Multidimensional trade-offs in crown functional trait trade-offs and principal component analysis (PCA) between shade tolerance guilds in the Dongbaishan forest.** (a) Spearman correlation matrix of crown traits, with line widths and colors scaled to correlation strength (see detailed specific *P*-values and *ρ*-values in Table S2). (b) PCA biplot of crown traits along the first two principal components (PC1 and PC2). Ellipses represent 95% confidence intervals for guild-specific centroids. Active variables (crown traits) are shown by solid black vectors; the supplementary variable (annual growth rate, AGR) is indicated by a dashed grey vector. Half-violin plots illustrate the distribution and divergence of PC1 and PC2 scores between guilds. Effect sizes (Cliff’s δ) are categorized as (absolute value): < 0.15 (negligible), 0.15–0.33 (small), 0.33–0.47 (medium), > 0.47 (large). Trait abbreviations: TH, tree height; CD, crown depth; LCR, live crown ratio; CAR, crown aspect ratio; ADR, apical dominance ratio; CPA, crown projection area. ∗*P* < 0.05, ∗∗*P* < 0.01, ∗∗∗*P* < 0.001.

Shade tolerance guilds exhibited distinct crown architectural strategies, with strong covariation among key traits. Crown depth (CD), live crown ratio (LCR), and crown aspect ratio (CAR) showed robust correlations (Spearman’s |*ρ*| > 0.6), with CD also positively correlated with crown projection area (CPA) (Fig. 2a and Table S2). Notably, CAR and CPA did not show significant covariation. Tree height (TH) and apical dominance ratio (ADR) were strongly positively associated (*ρ* > 0.6), whereas LCR showed weaker negative relationships with both TH and ADR (|*ρ*| < 0.6; Fig. 2a and Table S2). Controlling for size enhanced the CPA-ADR relationships but weakened the covariation between CD-CPA and TH-ADR (Table S2).

Principal component analysis (PCA) revealed two dominant axes collectively explaining over 70% of the variation. Specifically, CAR exhibited contrasting loadings on PC1 relative to CD and CPA, while TH and ADR loaded strongly together on PC2. Additionally, LCR exhibited substantial loadings on both axes (Table S3 and Fig. 2b). Both PC1 and PC2 showed significant associations with annual growth rate (AGR) (*P* < 0.001; Spearman’s *ρ* = 0.35 for PC1 and 0.57 for PC2; Fig. 2b). The guilds diverged strongly along PC2 (Cliff’s *δ* = 0.52), with shade-tolerant species exhibiting lower scores than light-demanding species (Fig. 2b). However, PC1 showed a significant but negligible separation between shade tolerance guilds (*δ* = −0.12; Fig. 2b).

### 3.2 Effects of crown trait dissimilarity on growth rate of shade tolerance guilds

**Fig. 3:**
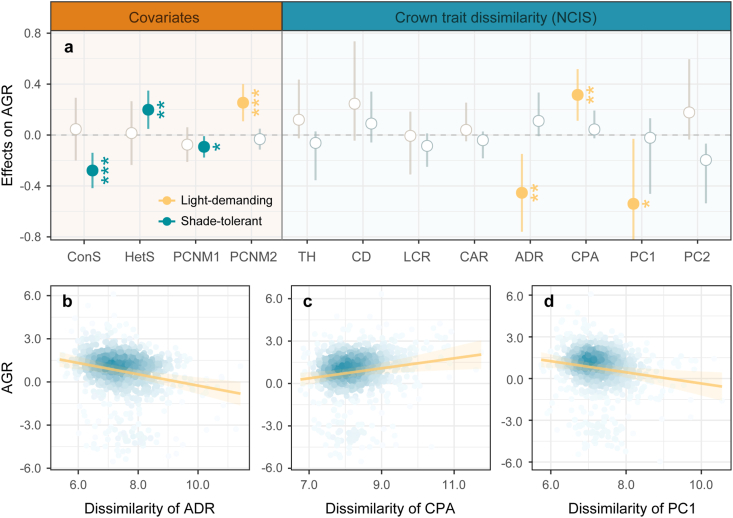
**Effects of covariates and crown trait dissimilarity (neighborhood crowding indexof trait dissimilarity,NCIS) on annual growth rate (AGR) from linear mixed-effects models (LMMs).** (a) Standardized coefficients (95% confidence intervals) from averaged best-fit models for light-demanding species (yellow, left) and shade-tolerant species (green, right). Solid symbols with darker hues represent statistically significant predictors (*P* < 0.05); open symbols with lighter hues indicate non-significant relationships. Only estimates and confidence intervals within [−0.8, 0.8] are shown for clarity. Covariates abbreviations: conspecific density, ConS; heterospecific density, HetS. Tait abbreviations follow Fig. 1. ∗*P* < 0.05, ∗∗*P* < 0.01, ∗∗∗*P* < 0.001. (b–d) Partial residual plots illustrating the relationship between crown trait dissimilarity and AGR for light-demanding species’ model with lowest predictors (see Table S6 for details). Both axes are log-transformed.

Neighborhood effects on growth differed fundamentally between shade tolerance guilds (Fig. 3 and Fig. S1; Tables S4 and S5). While tree size (log-transformed DBH) and spatial structure (PCNM1/PCNM2) significantly influenced growth in both guilds, their sensitivity to local neighborhood interactions varied markedly (Fig. 3; Tables S4 and S5).

Light-demanding species exhibited strong crown-mediated effects without evidence of density dependence (ConS/HetS). Their annual growth rate (AGR) increased with greater dissimilarity in crown projection area (CPA), but decreased with dissimilarity in apical dominance ratio (ADR) and crown shape (PC1). These patterns remained robust across both the model-averaged results and the minimal-predictor models (Fig. 3a–d and Table S6). In contrast, shade-tolerant species were inhibited by conspecific neighbors but facilitated by heterospecific neighbors, whereas no significant effects of crown trait dissimilarity were detected in the best-fit model (Fig. 3a).

## 4 Discussion

### 4.1 Difference in the crown trait trade-off strategies of shade tolerance guilds

Our analysis uncovered coordinated patterns in crown architecture allocation through examination of trait correlations. Crown depth (CD) was correlated with both crown aspect ratio (CAR) and crown projection area (CPA), but CAR and CPA showed no direct association (Fig. 2a). This suggests that crown expansion in one dimension may compensate for constraints in another, balancing whole-tree resource allocation under varying light regimes (Sugiura and Tateno, 2013). The absence of direct CAR-CPA covariation likely arises from species-specific allometric scaling in crown vertical and horizontal dimensions. Beyond the inherent horizontal-vertical crown trade-off, we identified a covariation among tree height (TH), apical dominance ratio (ADR), and live crown ratio (LCR) (Fig. 1a), indicating that taller trees may develop proportionally smaller crowns through apical control and foliage distribution adjustments (Maguire and Bennett, 1996).

Tree size (log-transformed DBH) mediated crown trait relationships (Table S3). Controlling for tree size strengthened the covariation between CPA and ADR, revealing a more intrinsic linkage between horizontal crown expansion and apical control mechanisms. Conversely, size correction weakened covariation between CD and CPA, and between TH and ADR, demonstrating that these initial relationships were driven by allometric scaling with tree size (Bohlman and O’Brien, 2006).

Principal component analysis delineated a two-dimensional adaptive space for crown traits (Fig. 2). The PC1 primarily represented variation in CD, LCR, CAR, and CPA (Fig. 2b), consistent with similar results from earlier research (Jackson et al., 2019). PC1 captured a continuum from “fast” strategies (narrow, deep crowns) to “slow” strategies (broad, shallow crowns), mirrored by its correlation with annual growth rate (AGR) (Fig. 2b). While this crown economic spectrum appears to parallel classical leaf-economic frameworks, certain crown traits have been identified as less phylogenetically conserved and more environmentally sensitive compared to organ traits (Maynard et al., 2022). This disparity suggests these spectra may operate through distinct mechanisms, potentially decoupling crown-level carbon acquisition strategies from organ-level investment patterns.

The PC2 was mainly associated increasing TH and ADR with reducing LCR, and also exhibited a relationship with AGR (Fig. 2a and c). This size-dependent trade-off in whole-plant morphology may result from taller trees developing smaller crowns to minimize damage risk (Gardiner et al., 2016), extending the previously described organ-size-based “stature-recruitment” trade-off to incorporate crown dimensions. Such architectural optimization resolves an ontogenetic dilemma: initial crown expansion maximizes light interception during pre-canopy stages, while post-canopy height growth necessitates crown streamlining to maintain positive carbon balance (Fransson et al., 2021). Mechanistically, this may involve a strategy to mitigate stress by accelerating the death and shedding of branches and leaves in the lower crown (Li et al., 2022). However, the extent to which our findings link with emerging “light-water” crown economic spectra remains unknown (McNeil et al., 2023), highlighting the need for further studies to integrate analyses of crown architecture, hydraulic traits, and light capture efficiency.

Shade tolerance guilds exhibited distinct trade-off strategies of crown traits (Fig. 3b and d), which aligns with previous research findings based on organ traits (Ameztegui et al., 2017). Contrary to the traditional hypothesis that shade-tolerant species develop wider, shallower crowns to reduce self-shading (Horn, 1971; but see Martin-Ducup et al., 2020; Owen et al., 2021), we observed no significant difference in PC1 scores (associated with narrow, elongated crowns) between shade-tolerant and light-demanding species (Fig. 2b). The lack of guild divergence on PC1 may stem from shade-tolerant species’ lower light compensation point (Poorter et al., 2003), permitting greater architectural flexibility in crown form without strict pressure to minimize self-shading. Light-demanding species occupied higher PC2 positions (Fig. 2b), adopting emergent-layer strategies with compact crowns to capitalize on transient canopy gaps. These divergent solutions of species with different shade tolerance to the crown optimization problem reinforce the role of architectural plasticity in species coexistence (Buche et al., 2022).

### 4.2 Differences in crown trait dissimilarity effects on growth rate of shade tolerance guilds

Guild-specific responses occurred despite consistent effects of tree size and spatial structure across all species. The effects of tree size reflect ontogenetic shifts in resource requirements, with larger trees typically dominating resource acquisition (Coomes and Allen, 2007). Spatial structure effects indicate microhabitat heterogeneity influences all species, likely through unmeasured edaphic or light gradients (Jones et al., 2008).

Our findings reveal fundamental differences in how crown-mediated neighborhood interactions influence growth dynamics across shade tolerance guilds (Fig. 3). Light-demanding species exhibited strong growth dependence on crown trait dissimilarity (Fig. 3b; Tables S4 and S5), consistent with their physiological sensitivity to light competition (Kothari et al., 2021). Specifically, apical dominance ratio (ADR) and PC1 value dissimilarity showing a significant negative relationship with AGR, while crown projection area (CPA) dissimilarity enhanced growth (Fig. 3 and Table S5). In contrast, shade-tolerant species showed no significant association between crown trait dissimilarity and AGR; instead, neighborhood density exerted dominant control over their growth (Fig. 3 and Table S4).

The growth suppression under high ADR dissimilarity may reflect the “light competition hypothesis” for apical dominance evolution, where this trait mitigates shading and enhances light interception from neighbors (Aarssen, 1995). This pattern is consistent with environmental filtering favoring trait convergence in light acquisition strategies, selecting against neighboring individuals with mismatched architectures (Lasky et al., 2013; Chen et al., 2016; Lusk and Laughlin, 2017). Similarly, reduced growth performance driven by PC1 dissimilarity may reflect environmental filtering on the crown shape syndrome. Conversely, increased dissimilarity in CPA enhanced AGR indicating that reduced crown horizontal overlap may lead to niche differentiation through complementary use of light resources (Sapijanskas et al., 2014; Zambrano et al., 2019), thereby reducing competition and even promoting facilitation among neighboring individuals (Bulleri et al., 2016).

Shade-tolerant species presented a distinctly opposed response pattern. One potential explanation for our results comes from the Janzen-Connell hypothesis, which predicts that the increase in conspecific density can lead to a higher risk from host-specific natural enemies like pathogens, parasites and herbivores (Wang et al., 2022; Hülsmann et al., 2024). Conversely, the growth-promoting effect of heterospecific density may reflect “species herd protection”, a phenomenon aligning with the concept of associational defense where increased heterospecific density dilutes enemy pressure (Peters, 2003). This decoupling of growth performance from light resource acquisition corroborates empirical evidence that shade-tolerant species experience greater pathogen pressure compared to light-demanding species (McCarthy-Neumann and Ibáñez, 2013).

Besides the ecological strategies of different shade tolerance guilds prioritizing either low-light survival over pathogen resistance or the reverse, distinct responses to topographic factors between guilds also imply their habitat preferences (Jin et al., 2018). Taken together, our findings emphasize that neglecting shade tolerance can obscure how individuals adjust and respond to their local environment, as this could impair our perception of individual-level performances. While our work provides trait-based insights into crown-mediated neighborhood interactions, some key limitations warrant attention. First, the absence of individual-level organ traits (e.g., leaf economic traits) precludes the relative importance of whole-plant trait-mediated interactions in driving growth performance. Second, given the environmental sensitivity and latitude dependency of crown traits (Aakala et al., 2016; Jucker et al., 2025), cross-biome studies explicitly comparing climatic zones could elucidate how crown-mediated ecological process maintain species coexistence across geographic gradients. Further studies combining organ-level traits (e.g., leaf morphology and hydraulic traits) and crown architecture across sites within large-scale forest monitoring networks such as CForBio and ForestGEO are needed to advance this framework.

## 5 Conclusions

This study establishes that crown architectural trade-offs and their interactions with neighborhood effects are intrinsically structured by species’ shade tolerance. By analyzing multidimensional crown traits and tree growth data from a subtropical forest, we identified two key axes of crown trait variation: a “crown shape” spectrum (PC1) reflecting gradients in light-harvesting efficiency, and a “crown size” spectrum (PC2) associated with potential for vertical stratification. Shade-tolerant species exhibit similar crown shapes to light-demanding species, reflecting that they may enhance resource capture in low light through flexible crown development. Conversely, light-demanding species optimize canopy dominance through compact crown geometries and accelerated height growth rates, facilitating more effective preemption of canopy space.

Crown trait dissimilarity and neighborhood density exerted guild-specific effects on growth. Notably, crown trait dissimilarity significantly mediated tree growth exclusively in light-demanding species, dissimilarity in apical dominance ratio was associated with reduced growth, whereas dissimilarity in crown projection area alleviated competitive interactions. In contrast, growth rates in shade-tolerant species were primarily influenced by local neighborhood density. These differential responses reveal that trait-mediated biotic interactions are context-dependent and closely linked species’ life history strategies. By linking crown-mediated neighborhood interactions to demographic outcomes, this study provides a mechanistic basis for integrating multidimensional crown trait organization and shade tolerance continua into forest dynamics models. Such integration is essential for accurately predicting mechanisms of species coexistence and successional trajectories under changing environmental regimes.

## CRediT authorship contribution statement

**Qi Wu**: Writing – review & editing, Writing – original draft, Methodology, Formal analysis, Visualization, Conceptualization. **Xingchen Wang**: Writing – original draft, Validation, Methodology, Visualization, **Yumeng Huang**: Writing – review & editing, Investigation, Data curation. **Chengwei Li**: Writing – review & editing, Investigation, Data curation. **Lisheng Yang**: Writing – review & editing, Investigation, Data curation. **Zijun Zeng**: Writing – review & editing, Investigation, Data curation. **Yishan Shi**: Writing – review & editing, Investigation, Data curation. **Jianhua Chen**: Writing – review & editing, Data curation, Supervision, Conceptualization. **Yunquan Wang**: Writing – review & editing, Validation, Methodology, Investigation, Data curation, Conceptualization.

## Declaration of competing interest

The authors declare that they have no known competing financial interests or personal relationships that could have appeared to influence the work reported in this paper.
