# Supplementary material for: Crown architecture traits mediate shade tolerance-dependent trade-offs and neighborhood interactions in a subtropical forest
Source: Plant Divers. 2026 Feb 9;48(4):791–9. doi: 10.1016/j.pld.2026.02.004 (PMC13424391; doi:10.1016/j.pld.2026.02.004)

**Appendix:**

**Table S1** Shade tolerance of the 31 species in the Dongbaishan Forest Plot.

| Species | Family | Light figure | Shade tolerance |
| --- | --- | --- | --- |
| *Liquidambar formosana* | Altingiaceae | 8 | Light-demanding |
| *Ilex rotunda* | Aquifoliaceae | 5 | Shade-tolerant |
| *Cunninghamia lanceolata* | Cupressaceae | 7 | Light-demanding |
| *Diospyros japonica* | Ebenaceae | 7 | Light-demanding |
| *Diospyros kaki* var. *silvestris* | Ebenaceae | 7 | Light-demanding |
| *Vaccinium mandarinorum* | Ericaceae | 6 | Shade-tolerant |
| *Rhododendron ovatum* | Ericaceae | 7 | Light-demanding |
| *Rhododendron simsii* | Ericaceae | 8 | Light-demanding |
| *Vaccinium bracteatum* | Ericaceae | 8 | Light-demanding |
| *Albizia kalkora* | Fabaceae | 7 | Light-demanding |
| *Dalbergia hupeana* | Fabaceae | 7 | Light-demanding |
| *Castanea seguinii* | Fagaceae | 7 | Light-demanding |
| *Lithocarpus glaber* | Fagaceae | 7 | Light-demanding |
| *Quercus acutissima* | Fagaceae | 7 | Light-demanding |
| *Quercus fabri* | Fagaceae | 7 | Light-demanding |
| *Quercus serrata* | Fagaceae | 7 | Light-demanding |
| *Castanopsis sclerophylla* | Fagaceae | 8 | Light-demanding |
| *Loropetalum chinense* | Hamamelidaceae | 8 | Light-demanding |
| *Lindera glauca* | Lauraceae | 7 | Light-demanding |
| *Sassafras tzumu* | Lauraceae | 8 | Light-demanding |
| *Morella rubra* | Myricaceae | 6 | Shade-tolerant |
| *Eurya muricata* | Pentaphylacaceae | 6 | Shade-tolerant |
| *Pinus massoniana* | Pinaceae | 9 | Light-demanding |
| *Rhaphiolepis indica* | Rosaceae | 5 | Shade-tolerant |
| *Gardenia jasminoides* | Rubiaceae | 5 | Shade-tolerant |
| *Schoepfia jasminodora* | Schoepfiaceae | 6 | Shade-tolerant |
| *Styrax dasyanthus* | Styracaceae | 7 | Light-demanding |
| *Styrax odoratissimus* | Styracaceae | 7 | Light-demanding |
| *Symplocos sumuntia* | Symplocaceae | 6 | Shade-tolerant |
| *Camellia fraterna* | Theaceae | 7 | Light-demanding |
| *Schima superba* | Theaceae | 6 | Shade-tolerant |

**Table S2** Spearman correlations (below diagonal) and DBH-weighted partial Spearman correlations (above diagonal) for tree crown traits. TH, tree height; CD, crown depth; LCR, live crown ratio; CAR, crown aspect ratio; ADR, apical dominance ratio; CPA, crown projection area.

|  | TH | CD | LCR | CAR | ADR | CPA |
| --- | --- | --- | --- | --- | --- | --- |
| TH | - | 0.06*** | -0.23*** | -0.13*** | 0.48*** | -0.02 |
| CD | 0.47*** | - | **0.90***** | **-0.77***** | -0.28*** | 0.49*** |
| LCR | -0.31*** | **0.62***** | - | **-0.71***** | -0.47*** | 0.43*** |
| CAR | -0.18*** | **-0.71***** | **-0.65***** | - | -0.23*** | 0.11*** |
| ADR | **0.61***** | 0.02 | -0.51*** | -0.26*** | - | -0.75*** |
| CPA | 0.51*** | **0.66***** | 0.19*** | 0.00 | -0.25*** | - |

**Table S3** Summary statistics of tree crown architecture traits, including mean, standard deviation (SD), coefficient of variation (CV), and loadings on the first two principal components (PC1 and PC2). Trait abbreviations are described in Table S2.

| Trait | Mean | SD | CV (%) | PC1 | PC2 |
| --- | --- | --- | --- | --- | --- |
| TH | 8.58 | 3.53 | 41.09 | 0.38 | 0.87 |
| CD | 3.10 | 2.02 | 64.98 | 0.95 | 0.13 |
| LCR | 0.38 | 0.20 | 51.06 | 0.75 | -0.55 |
| CAR | 0.99 | 0.75 | 75.69 | -0.58 | -0.11 |
| ADR | 3.98 | 2.32 | 58.43 | -0.24 | 0.79 |
| CPA | 5.00 | 4.19 | 83.84 | 0.66 | 0.13 |

**Table S4** The models for the growth rate of shade tolerance guilds with a difference in AIC (ΔAIC) of ≤ 2 compared to the model with the smallest AIC are shown. Covariates are omitted here for all best-fit models shared the same. Trait abbreviations are described in Table S2; here refer to the neighborhood crowding index weighted by trait dissimilarity (NCIS) for the corresponding traits.

| Explanatory variable | df | ΔAIC |
| --- | --- | --- |
| Light-demanding | | |
| TH+CD+ADR+CPA+PC1 | 14 | 0.00 |
| TH+CD+ADR+CAR+CPA+PC1+PC2 | 15 | 0.32 |
| CD+ADR+CPA+PC1+PC2 | 14 | 0.53 |
| TH+CD+ADR+CAR+CPA+PC1 | 15 | 0.89 |
| TH+CD+ADR+CPA+PC1+PC2 | 15 | 0.91 |
| TH+ADR+CPA+PC1 | 13 | 0.92 |
| ADR+CPA+PC1+PC2 | 13 | 1.32 |
| TH+ADR+CPA+PC1+PC2 | 14 | 1.46 |
| TH+CD+ADR+CAR+CPA+PC1+PC2 | 16 | 1.48 |
| ADR+CAR+CPA+PC1+PC2 | 14 | 1.57 |
| CD+ADR+CAR+CPA+LCR+PC1+PC2 | 16 | 1.88 |
| TH+CD+ADR+CPA+LCR+PC1 | 15 | 1.98 |
| Shade-tolerant | | |
| CD+LCR+ADR+CAR+PC2 | 14 | 0.00 |
| TH+LCR+CPA | 12 | 0.56 |
| ADR+CPA+PC2 | 12 | 0.57 |
| ADR+PC2 | 11 | 0.73 |
| ADR+CAR+CPA+PC2 | 13 | 0.82 |
| TH+CD+LCR+CAR | 13 | 0.82 |
| TH+CD+LCR | 12 | 0.93 |
| TH+CD+LCR+CPA+PC1 | 14 | 1.11 |
| TH+CD+LCR+CPA | 13 | 1.12 |
| ADR+LCR+CPA+PC2 | 13 | 1.18 |
| CD+ADR+CAR+PC2 | 13 | 1.21 |
| ADR+CAR+PC2 | 12 | 1.29 |
| TH+CD+LCR+CAR+CPA | 14 | 1.34 |
| CD+ADR+LCR+PC2 | 13 | 1.36 |
| CD+ADR+CPA+LCR+PC1+PC2 | 15 | 1.36 |
| TH+CD+ADR+LCR+CAR | 14 | 1.59 |
| TH+CD+ADR+LCR+CAR+PC2 | 15 | 1.67 |
| ADR+CAR+LCR+CPA+PC2 | 14 | 1.78 |
| CD+ADR+LCR+CAR+PC1+PC2 | 15 | 1.80 |
| TH+LCR+CAR+CPA | 13 | 1.92 |
| CD+ADR+LCR+CPA+PC2 | 14 | 1.94 |
| CD+LCR+CAR | 12 | 1.94 |
| TH+ADR+CPA+PC2 | 13 | 1.95 |
| CD+ADR+CAR+CPA+LCR+PC1+PC2 | 16 | 1.97 |
| CD+ADR+PC2 | 12 | 1.97 |

**Table S5** Averaged best-fit models predicting growth rate for light-demanding and shade-tolerant species guilds. Covariates include log-transformed diameter at breast height (log(DBH)), conspecific density (ConS), heterospecific density (HetS), spatial eigenvectors (PCNM1, PCNM2), and neighborhood crowding indices weighted by trait dissimilarity (NCIS; abbreviations defined in Table S2). Estimates, 95% confidence intervals (95% CI), and standard errors (SE) are shown. **P* ≤ 0.05, ***P* ≤ 0.01, ****P* ≤ 0.001.

| Variable | Estimate | 95%CI |  | SE |
| --- | --- | --- | --- | --- |
|  |  | 2.5% | 97.5% |  |
| Light-demanding | | | | |
| log(DBH) | **2.12***** | 1.90 | 2.35 | 0.11 |
| ConS | 0.05 | -0.20 | 0.29 | 0.13 |
| HetS | 0.02 | -0.24 | 0.27 | 0.13 |
| PCNM1 | -0.08 | -0.21 | 0.06 | 0.07 |
| PCNM2 | **0.25***** | 0.11 | 0.40 | 0.07 |
| TH | 0.12 | -0.03 | 0.43 | 0.13 |
| CD | 0.25 | -0.04 | 0.74 | 0.23 |
| LCR | -0.01 | -0.31 | 0.18 | 0.05 |
| CAR | 0.04 | -0.05 | 0.25 | 0.07 |
| ADR | **-0.45**** | -0.76 | -0.15 | 0.16 |
| CPA | **0.32**** | 0.11 | 0.52 | 0.10 |
| PC1 | **-0.54*** | -1.05 | -0.03 | 0.26 |
| PC2 | 0.18 | -0.04 | 0.60 | 0.19 |
| Shade-tolerant | | | | |
| log(DBH) | **1.96***** | 1.83 | 2.10 | 0.07 |
| ConS | **-0.28***** | -0.42 | -0.14 | 0.07 |
| HetS | **0.20**** | 0.05 | 0.35 | 0.08 |
| PCNM1 | **-0.09*** | -0.18 | -0.01 | 0.04 |
| PCNM2 | -0.03 | -0.11 | 0.05 | 0.04 |
| TH | -0.06 | -0.36 | 0.03 | 0.10 |
| CD | 0.09 | -0.06 | 0.34 | 0.11 |
| LCR | -0.09 | -0.25 | 0.01 | 0.08 |
| CAR | -0.04 | -0.18 | 0.03 | 0.05 |
| ADR | 0.11 | -0.01 | 0.33 | 0.10 |
| CPA | 0.04 | -0.03 | 0.19 | 0.06 |
| PC1 | -0.02 | -0.46 | 0.13 | 0.08 |
| PC2 | -0.20 | -0.54 | -0.07 | 0.17 |

**Table S6** Parameter estimates for growth models of light-demanding species with lowest predictors. See Methods and Table S5 for covariate (log(DBH), ConS, HetS, PCNM, NCIS traits) and model details. Estimates, 95% confidence intervals (95% CI), and standard errors (SE) are shown. **P* ≤ 0.05, ***P* ≤ 0.01, ****P* ≤ 0.001.

| Variable | Estimate | 95%CI |  | SE | VIF |
| --- | --- | --- | --- | --- | --- |
|  |  | 2.5% | 97.5% |  |  |
| Top-ranked model (corresponds to Figure 2) | | | | |  |
| log(DBH) | **2.14***** | 1.92 | 2.37 | 0.11 | 1.23 |
| ConS | 0.04 | -0.20 | 0.28 | 0.12 | 1.65 |
| HetS | 0.03 | -0.22 | 0.27 | 0.13 | 2.71 |
| PCNM1 | -0.07 | -0.20 | 0.06 | 0.07 | 1.15 |
| PCNM2 | **0.24***** | 0.10 | 0.39 | 0.07 | 1.32 |
| TH | **0.26**** | 0.07 | 0.45 | 0.10 | 2.26 |
| CD | - | - | - | - |  |
| LCR | - | - | - | - |  |
| CAR | - | - | - | - |  |
| ADR | **-0.35***** | -0.53 | -0.18 | 0.09 | 2.65 |
| CPA | **0.25**** | 0.06 | 0.44 | 0.10 | 3.17 |
| PC1 | **-0.26**** | -0.45 | -0.07 | 0.10 | 3.22 |
| PC2 | - | - | - | - |  |
| Alternative model (second highest AIC weight) | | | | |  |
| log(DBH) | **2.11***** | 1.89 | 2.33 | 0.11 | 1.16 |
| ConS | 0.12 | -0.12 | 0.35 | 0.12 | 1.56 |
| HetS | 0.10 | -0.14 | 0.34 | 0.12 | 2.48 |
| PCNM1 | -0.08 | -0.21 | 0.06 | 0.07 | 1.15 |
| PCNM2 | **0.23**** | 0.08 | 0.37 | 0.07 | 1.30 |
| TH | - | - | - | - |  |
| CD | - | - | - | - |  |
| LCR | - | - | - | - |  |
| CAR | - | - | - | - |  |
| ADR | **-0.60***** | -0.83 | -0.36 | 0.12 | 4.86 |
| CPA | **0.31***** | 0.12 | 0.49 | 0.09 | 2.95 |
| PC1 | **-0.28**** | -0.46 | -0.09 | 0.10 | 3.22 |
| PC2 | **0.34**** | 0.09 | 0.60 | 0.13 | 5.35 |

**Fig. S1.** Log-transformed annual growth rates (AGR) of shade tolerance guilds.


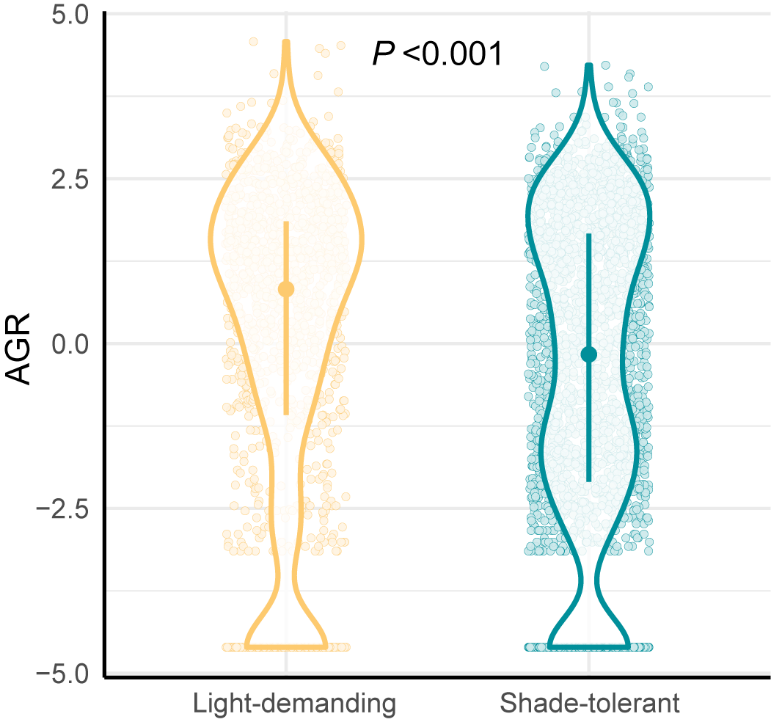

Supplement: Multimedia component 1 [file mmc1.docx]
